# Differentially enriched fungal communities in root rot resistant and susceptible varieties of tobacco (*Nicotiana tabacum L*.) under continuous monoculture cropping

**DOI:** 10.3389/fmicb.2022.1036091

**Published:** 2022-12-07

**Authors:** Jincheng Ao, Zheng Wang, Qigang Yang, Bo Li, Ying Li, Yongmei Li

**Affiliations:** ^1^College of Plant Protection, Yunnan Agricultural University, Kunming, China; ^2^Yunnan Tuer Lanyi Agricultural Technology Co., Ltd., Kunming, China; ^3^China Tobacco Guangxi Industrial Co., Ltd., Nanning, China

**Keywords:** *Nicotiana tabacum*, root rot, fungal diversity, rDNA gene sequencing, physicochemical properties

## Abstract

Root rot is a major disease of tobacco that causes crop losses of up to 15–20% of global tobacco production. The present study aimed to compare the fungal communities, and physicochemical properties of rhizosphere soil of root rot resistant (Yunyan 87; Y) and susceptible (*Honghua Dajinyuan*; H) tobacco varieties. Four treatments of each variety under continuous monocropping cultures included: control groups (HT0 and YT0); 2 years of continuous cropping (HT2 and YT2); 4 years of continuous cropping (HT4 and YT4); and 8 years of continuous cropping (YT8 and HT8). The soil physicochemical properties including available nitrogen (AN), available phosphorus (AP), available potassium (AK), and organic matter (OM) were increased (*p* < 0.05) from HT0 to HT8, whereas the resistant variety (Y) showed an inconsistent trend from YT0 to YT8. The pH was decreased (*p* < 0.05) from HT0 to HT8 and YT0 to YT8. Further, the disease incidence rate and disease index of the H variety also increased (*p* < 0.05) from HT0 to HT8. Alpha diversity analysis revealed that susceptible variety had higher fungal diversity from HT0 to HT8, while resistant variety exhibited lower diversity from YT0 to YT8. Ascomycota and Mortierellomycota were the dominant phyla in H and Y. Ascomycota abundance was increased (*p* < 0.05), whereas Mortierellomycota was decreased (*p* < 0.05) for continuous cropping years in H and Y. Penicillium, Fusarium, and Chrysosporium were the top three abundant genera in both varieties. The relative abundance of Penicillium spp. was increased (*p* < 0.05) in Y, whereas decreased (*p* < 0.05) in H variety. Specifically, Chrysosporium spp. was increased (*p* < 0.05) whereas Fusarium spp. was decreased (*p* < 0.05) in YT2. Redundancy analysis (RDA) revealed that fungal communities in H and Y rhizospheres were influenced by pH and carbon content, respectively. The top three highly enriched (*p* < 0.05) pathways in both varieties were fatty acid elongation, fatty acid β-oxidation I, and glyoxylate cycle. Our study concluded that resistant variety exhibited lower fungal diversity and functionally enriched metabolic pathways than susceptible variety that might be the result of molecular breeding practices, however, the relative abundance of Penicillium spp. were increased in resistant variety under long-term monoculture cropping.

## Introduction

Root rots have a considerable influence on crop productivity worldwide (Kumari and Katoch, 2020). Crop losses can vary from just beyond the economic threshold to losing whole fields, depending on the causative agent, host vulnerability, and climatic conditions (Williamson-Benavides and Dhingra, 2021). Fungi and oomycetes are the most prevalent pathogens observed in root rot disease. Fungal species like *Rhizoctonia solani* and mostly belonging to the genus Fusarium are more common causative agents (Chaudhary et al., 2021). Bacteria and even viruses, on the other hand, might also be directly involved in onset of root rot (Williamson-Benavides and Dhingra, 2021).

Tobacco (*Nicotiana tabacum L.*) is an economically important crop grown in over 125 countries across the world. China ranks first in tobacco leaves production, with an estimated annual yield of over 2.2 million tons (Wang et al., 2022b). Fusarium spp. causing tobacco root rot disease is one of the most prevalent and widespread soil-borne diseases, frequently co-occurring with tobacco bacterial wilt and black shank diseases (Ma et al., 2018). Fusarium root rot resulting from *Fusarium solani* species complex (FSSC) and *Fusarium oxysporum* species complex (FOSC) is common (Abd-El-Khair et al., 2019; Ma et al., 2019; Gibert et al., 2022). Chlorosis and wilt are disease indicators that spread from the bottom to the top leaves. Members of FOSC and FSSC have been associated with tobacco wilt and root rot in China (Yang et al., 2020a; Gai et al., 2021) and other tobacco-growing regions worldwide (Berruezo et al., 2018; Abd-El-Khair et al., 2019). FSSC and FOSC pathogens are responsible for crop losses of up to 15–20% of global tobacco production.

As soil-borne diseases are difficult to control, it is crucial to apply integrated management measures. The development and cultivation of root rot resistant or tolerant tobacco cultivars are one of the efficient methods of controlling Fusarium wilt and root rot (Sánchez-García and Mora Avilés, 2022). Soil microorganisms play a significant role in plant stress resilience, resistance to soil-borne diseases, nutrient absorption capacity, and host immune regulation (Zhang et al., 2017; Korenblum et al., 2020). Selective breeding can shape the rhizosphere microbial communities and change their diversity (Favela et al., 2021). Succession pattern of changes in soil microbiome can be investigated through metagenomic sequencing which is an advanced technique to characterize the structure and diversity of microbial communities. Metagenomic studies can help to better understand the underlying mechanisms of disease development by exploring changes in the microbial diversity in healthy and diseased plant soils. Furthermore, many studies have been conducted to explore the bacterial and fungal communities structure in rhizosphere soil by sequencing 16S rRNA genes and internal transcribed spacer (ITS) region, respectively (BenIsrael et al., 2021; Tran, 2022).

A continuous cropping system is cultivation of the same or similar crop in the same soil year after year (Yang et al., 2020b). Continuous cropping might result in alteration in the microbial community structure of soil rhizosphere, nutrient imbalance, and autotoxicity of root exudates (Zhu et al., 2018). Long-term continuous cropping, on the other hand, frequently results in increased growth of soil-borne plant pathogens and decreased crop production, which is termed as continuous cropping obstacle (Tan et al., 2021). The previous studies have revealed that continuous cropping resulted in the disturbance of the rhizosphere soil microbiome and decreased production of many important economical crops like soybean (Tian et al., 2020), sweet potato (Gao et al., 2019), and cotton (Xi et al., 2019). In China, however, continuous cropping is popular with diverse agricultural systems due to limited arable land and inadequate farming methods (Lei et al., 2020). Under this scenario, it is imperative to investigate the relationship and mechanisms involved in continuous monoculture farming and soil microbial ecosystems.

The aim of the present study was to examine differences in soil physicochemical properties, fungal community structure, and functional enrichment of metabolic pathways in two tobacco varieties *Yunyan 87* (root rot resistant) and *Honghua Dajinyuan* (root rot susceptible) under continuous cropping cultures. The fungal communities in the rhizosphere soil of both varieties were compared and analyzed by the Illumina Miseq high throughput sequencing. The findings of present study provide practical insights into an in-depth understanding of the microbial mechanism of the continuous cropping obstacle of *N. tabacum L.* cultivars and would help in the prevention and control of root rot.

## Materials and methods

### Experimental site location and soil properties

The present experiment was conducted in pots by growing the two varieties of tobacco (*N. tabacum L.)* plant, *Yunyan 87* (root rot resistance variety) (Rui et al., 2019), and *H. Dajinyuan* (root rot susceptible variety) in Malong district, Yunnan province, China (25^°^21′ N, 103^°^23′ E). Samples of *Yunyan 87* were labeled as “Y” and *H. Dajinyuan* was labeled as “H.” On 5 June 2021, the floating seedling method was used to grow the seedlings in the greenhouse. The tobacco plants were grown until the stage of four leaves and one heart in the greenhouse and two leaves were clipped. On 10 August 2021, they were transplanted into soils of different continuous cropping years (control group [0y], 2 years continuous cropping soil [2y], 4 years continuous cropping soil [4y], and 8 years continuous cropping soil [8y]), and watered enough to fix the roots. Plastic flower pots were utilized with an upper diameter of 33 cm, a lower diameter of 17 cm, and a height of 21 cm. Each pot contained 15.0 ± 0.5 kg of soil. The completely randomized design (CRD) was applied with two varieties *Yunyan 87* (Y) and *H. Dajinyuan* (H), each variety had four treatments (*Yunyan 87* [Y]: YT0, control group; YT2, plants transplanted in 2 years continuous cropping rhizosphere soil; YT4, plants transplanted in 4 years continuous cropping rhizosphere soil, and YT8, plants transplanted in 8 years continuous cropping rhizosphere soil and, *H. Dajinyuan* [H]: HT0, control group; HT2, plants transplanted in 2 years continuous cropping rhizosphere soil; HT4, plants transplanted in 4 years continuous cropping rhizosphere soil, and HT8, plants transplanted in 8 years continuous cropping rhizosphere soil). Moreover, each treatment was replicated four times and each replicate had one pot sample (Supplementary Table 1). While transplanting, a special compound fertilizer [m(N):m(P_2_O_5_):m(K_2_O) = 12:10:24] at 36 g/plant, and commercial organic fertilizer (organic matter [OM] ≥ 45%, total nutrients ≥ 5%) at 200 g/plant were applied. Potassium nitrate [m(N):m(K_2_O) = 13.5:44.5] at 9.0 g/plant was applied in rings and covered with soil for seedling raising.

The rhizosphere soil samples were collected from each treatment on the 65th day after transplantation. After removing sundries from pots, whole tobacco plants were removed from pots gently. Then, the peripheral soil of the root system was removed and rhizosphere soil within 2 mm of the fibrous root was collected by gently shaking it. A part of the rhizosphere soil was brought back to the laboratory for natural air-drying and passed through a 100-mesh sieve for analysis of physicochemical properties. Similarly, a part of the soil samples from each treatment replicate was put into a 50 ml sampling centrifuge tube and quickly put into an ice box, brought back to the lab, and stored at –80^°^C to be used for high-throughput sequencing later.

### Soil physicochemical properties

Soil suspension in water (1:2.5 WV^–^) was made to determine the pH of the soil using a pH meter (PHS-3C, INESA Scientific Instrument Co., Ltd., Shanghai, China). Hydrochloric acid and ammonium quantify available phosphorus (AP) according to the Molybdenum Blue technique (Watanabe and Olsen, 1965). Alkaline hydrolyzable diffusion was used to determine the amount of available nitrogen (AN) (Dodor and Tabatabai, 2019). Additionally, ammonium acetate was used to extract and quantify the available potassium (AK) using flame photometry. Soil OM was determined by redox titration with 0.8 mol/L K_2_Cr_2_O_7_. Fresh soil samples were used to extract the NH_4_^+^-N and N0_3_^–^-N from the soil and quantified using the SmartChem140 Automatic Chemical Analyzer (Pang et al., 2021).

### Disease incidence rate and disease index of *Honghua Dajinyuan* (Susceptible variety)

For disease incidence rate and disease index of *H. Dajinyuan* (susceptible variety), a CRD was applied with three treatments (HT2: 2 years continuous cropping plants; HT4: 4 years continuous cropping plants and; HT8: 8 years continuous cropping plants) with three replicates each. A total of 200 plants from each replicate were selected and were classified into grades (1–9) depending upon disease severity according to the Grade and Investigation Method of Tobacco Diseases and Insect Pests (GB/T 23222-2008) (Kong et al., 2009). The incidence rate and disease index were calculated by using the five spot sampling method. Following formulas were used for the calculation of disease incidence rate and disease index:


$$\mathrm{Disease}\mathrm{incidence}\mathrm{rate}\left(\%\right)=\frac{\mathrm{number}\,\mathrm{of}\,\mathrm{susceptible}\,\mathrm{plants}}{\mathrm{total}\,\mathrm{number}\,\mathrm{of}\,\mathrm{investigated}\,\mathrm{plants}}\times 100$$

$$\mathrm{Disease}\mathrm{Index}\left(\%\right)=\Sigma \,\frac{\begin{array}{c} (\mathrm{representative}\mathrm{value}\mathrm{of}\mathrm{disease}\mathrm{level}\\ \times \mathrm{number}\mathrm{of}\mathrm{samples}\mathrm{of}\mathrm{this}\mathrm{level}) \end{array}}{\begin{array}{c} (\mathrm{representative}\mathrm{value}\mathrm{of}\mathrm{the}\mathrm{highest}\mathrm{level}\\ \times \mathrm{total}\mathrm{number}\mathrm{of}\mathrm{samples}) \end{array}}\times 100$$

### Deoxyribonucleic acid extraction and internal transcribed spacer amplicon sequencing

The genomic deoxyribonucleic acid (DNA) of the rhizosphere soil samples was extracted using the MN NucleoSpin 96 SOI kit (Omega Bio-tek, Norcross, GA, USA), and then the purity and concentration of the DNA were evaluated by 1% agarose gel electrophoresis. The diluted genomic DNA was sequenced by Illumina MiSeq amplicon sequencing using primers labeled with Barcodes: ITS1 F, 5′-CTTGGTCATTTAGAGGAAGTAA-3′ (Gardes and Bruns, 1993); ITS2 R, 5′-GCTGCGTTCTTCATCGATGC-3′ (White et al., 1990), to the fungal ITS1–ITS2 regions. Using a 96-well PCR machine (AB), the obtained PCR products were purified by 2% agarose gel electrophoresis by using the Monarch DNA gel extraction kit (New England Biolabs, MA, USA). The library was constructed using the TruSeq DNA PCR-Free Library Preparation Kit from Illumina Company, and then Qubit was used for quantification and library detection. After passing the test, NovaSeq 6000 was used for on-machine sequencing. High-throughput sequencing was performed by Beijing Guoke Biotechnology Co., LTD, Beijing, China.

### Statistical and bioinformatics analyses of sequence data

The sequenced amplicons were subjected to the Cutadapt V1.9.1 software (accessed on April 15th, 2022)^1^ for assessing the quality of data, and low-quality reads were removed. Further, the UCHIME algorithm was applied in USEARCH to obtain the clean reads and remove the chimera sequences (accessed on 15 April, 2022)^2^ (Edgar et al., 2011). By using the Uparse software V7.0.1001 (accessed on 15 April, 2022)^3^ the qualified reads were grouped into operational taxonomic units (OTUs) at 97% similarity (Yang et al., 2018). For each taxonomic level (kingdom, phylum, class, order, family, genus, and species), species annotation analysis was carried out using the Mothur software against the SILVA132 SSUrRNA database (accessed on 16 April, 2022)^4^ with the threshold set at 0.8^–1^, taxonomic data was collected and analyzed independently for each classification level. Multiple sequence alignment (MSA) of all representative sequences was performed in MUSCLE Version 3.8.31 (accessed on 16 April, 2022)^5^ software, and phylogeny was established (Edgar, 2004). The Qiime software (Version 1.9.1) was used to perform the alpha diversity (Observed-species, ACE, Shannon, Simpson, Good’s-coverage, Chao1) and beta diversity analyses (Unifrac distance, UPGMA clustering, Principal component analysis [PCA], PCoA, and Non-Metric Multi-Dimensional Scaling [NMDS]) analysis and the results were further analyzed with R software (Version 2.15.3) (accessed on 20 April, 2022).^6^ Using default parameters and a threshold LDA score of 4, Linear discriminant analysis effect size (LEfSe) software was used to identify biomarker taxa in both treatment groups. In R software, a permutation test was performed to obtain *p*-value for the Metastats analysis. The *p*-value was then adjusted using the Benjamini and Hochberg False Discovery Rate technique, yielding a *q*-value (White et al., 2009). The RDA function in the vegan package of R programme was used to determine the correlation of environmental variables. Functional analysis was performed by using the PICRUSt2 (Douglas et al., 2020) software by comparing the species composition information and functional differences between different samples or groups. Further, reads annotation was applied to create functional profiles searching against the Clusters of Orthologous Groups of proteins (COG) database (Tatusov et al., 2003).

## Results

### Physicochemical properties of rhizosphere soil

The physicochemical properties like AN, AP, AK, OM, and pH of rhizosphere soil of both varieties (H and Y) were analyzed (Table 1). Results revealed that AN, AP, and AK were increased significantly (*p* < 0.05) from HT0 to HT8 for H variety, while pH decreased from HT0 to HT8. The OM of soil increased up to 4 years of continuous cropping (from HT0 to HT4) but later on decreased after 4 years (HT8). The continuous cropping of resistant variety (Y) showed an inconsistent trend for these parameters. The AP and AK contents were increased (*p* < 0.05) from YT0 to YT4 and then decreased (*p* < 0.05) at YT8. However, OM and pH were decreased (*p* < 0.05) from YT0 to YT8, and AN improved (*p* < 0.05) from YT0 to YT8.

**TABLE 1:** Physicochemical properties (Mean ± SD) of both varieties *Honghua Dajinyuan* (H) and *Yunyan 87* (Y).

Treatments	Available nitrogen (AN) (m/kg)	Available phosphorus (AP) (m/kg)	Available potassium (K) (m/kg)	Organic matter (OM) (g/kg)	pH
HT0	31.60 ± 3.46^c^	37.45 ± 2.54^d^	310.90 ± 7.31^c^	26.65 ± 1.80^ab^	5.30 ± 0.18^a^
HT2	54.30 ± 4.95^ab^	40.0 ± 0.81^cd^	436.57 ± 19.69^b^	28.25 ± 1.50^ab^	5.1 ± 0.08*a*^bc^
HT4	52.78 ± 6.66^ab^	47.98 ± 3.83^bc^	483.60 ± 14.27^a^	29.23 ± 1.63^a^	4.9 ± 0.16^bc^
HT8	61.75 ± 11.62^a^	51.075 ± 6.98^b^	501.52 ± 23.23^a^	26.0 ± 0.18^ab^	4.8 ± 0.14^c^
YT0	41.13 ± 3.38^bc^	49.25 ± 2.20^bc^	289.0 ± 19.91^c^	26.65 ± 2.47^ab^	5.32 ± 0.13^a^
YT2	52.18 ± 9.48^ab^	45.33 ± 5.41^bcd^	427.13 ± 26.230^b^	28.78 ± 5.32^ab^	5.2 ± 0.08^ab^
YT4	46.85 ± 3.19^abc^	66.05 ± 5.34^a^	483.35 ± 14.02^a^	26.9 ± 1.41^ab^	5.18 ± 0.05^ab^
YT8	51.40 ± 4.12^ab^	51.25 ± 1.11^b^	470.38 ± 23.36^ab^	23.35 ± 0.73^b^	4.95.21^bc^

Values with different superscripts in the same column differ significantly (*P*-value < 0.05).

### Disease incidence rate and disease index of *Honghua Dajinyuan* (Susceptible variety)

The disease incidence rate and disease index in *H. Dajinyuan* (susceptible variety) were calculated (Table 2). Both disease incidence rate and disease index were significantly (*p* < 0.05) increased with continuous cropping years and were observed highest for HT8 (19 ± 3.38 and 8.43 ± 0.86, respectively).

**TABLE 2:** Disease incidence and disease index rate of *Honghua Dajinyuan* (susceptible variety).

*Honghua Dajinyuan*	No. of plants observed	No. of susceptible plants	Disease incidence rate (%)	Disease index (%)
2 years continuous cropping plants (HT2)	200	7.67 ± 1.76^b^	3.83 ± 0.88^b^	1.50 ± 0.17^b^
4 years continuous cropping plants (HT4)	200	17.67 ± 1.76^b^	8.83 ± 0.88^b^	3.97 ± 0.57^b^
8 years continuous cropping plants (HT8)	200	38 ± 6.56^a^	19 ± 3.38^a^	8.43 ± 0.86^a^

Values with different superscripts in the same column differ significantly (*P*-value < 0.05).

### Operational taxonomic units and annotation of fungal communities

The Illumina Miseq sequencing resulted in an average of 100,152 raw reads per sample (a total of 32 samples), and 87,575 (87.4%) clean reads were obtained after quality control. Further, an average of 81,584 (81.4%) effective tags per sample were utilized for OTUs assignment. A total of 3,243 OTUs were annotated to different taxonomic levels (phylum to species) for all samples and 284 OTUs were common among them (Supplementary Figures 1A,B).

### Alpha diversity analysis

The dilution curve revealed that each sample was equally enriched with fungal communities and sequencing depth was enough for diversity analysis (Supplementary Figure 2A). Further, the rank abundance curve revealed the uniform distribution of fungal communities in each sample (Supplementary Figure 2B). The alpha diversity matrices (ACE, Chao1, Simpson index, and Shannon index) revealed significant (*p* < 0.05) differences in the diversity of fungal communities between rhizosphere of both varieties (H and Y) (Table 3). Overall, the rhizosphere of susceptible variety (H) had higher values of alpha diversity indices and more significant differences in fungal communities from HT0 to HT8 than the Y rhizospheres (YT0 to YT8).

**TABLE 3:** Alpha diversity matrices (Mean ± SD) of each treatment group of both varieties *Honghua Dajinyuan* (H) and *Yunyan 87* (Y).

Treatments	Observed OTUs	ACE	Chao1	Simpson index	Shannon index
HT0	267.25 ± 8.1394^ab^	298.32 ± 12.29^ab^	305.46 ± 14.98^ab^	0.9142 ± 0.04^a^	5.2203 ± 0.46^a^
HT2	191.75 ± 15.80^cd^	265.98 ± 26.09^abc^	258.37 ± 19.10^abc^	0.4234 ± 0.09^d^	2.0831 ± 0.46^c^
HT4	258.50 ± 30.93^ab^	317.14 ± 38.80^a^	321.43 ± 39.32^a^	0.7327 ± 0.20^abc^	3.9640 ± 1.43^ab^
HT8	275.75 ± 24.55^a^	321.44 ± 37.47^a^	315.02 ± 45.05^ab^	0.8732 ± 0.05^ab^	4.7285 ± 0.31^a^
YT0	218.25 ± 29.39^bc^	288.03 ± 32.076^ab^	275.65 ± 30.06^abc^	0.6701 ± 0.06^bc^	3.0792 ± 0.50^bc^
YT2	162.75 ± 20.02^cd^	215.06 ± 26.48^c^	203.19 ± 25.54^c^	0.5584 ± 0.04^cd^	2.2208 ± 0.31^c^
YT4	160.25 ± 31.92^d^	239.00 ± 30.63^bc^	226.44 ± 38.66^c^	0.5446 ± 0.08^cd^	2.1650 ± 0.49^c^
YT8	179.25 ± 21.19^cd^	254.97 ± 32.06^abc^	241.28 ± 34.42^bc^	0.7142 ± 0.11^abc^	2.9569 ± 0.50^bc^

Values with different superscripts in the same column differ significantly (*P*-value < 0.05).

### Relative abundance of fungal communities

The relative abundance of fungal communities in each treatment group of both varieties (H and Y) was observed at phylum and genus (Tables 4, 5). At the phylum level, the top three most abundant phyla were Ascomycota, Mortierellomycota, and uncharacterized (unidentified) fungi in both (H and Y) rhizospheres (Table 4). The relative abundance of Ascomycota fungi was increased significantly (*p* < 0.05) in both varieties in HT8 and YT8 as compared to their controls HT0 and YT0, respectively. However, the relative abundance of Mortierellomycota was decreased significantly (*p* < 0.05) in both varieties at HT8 and YT8 as compared to their controls HT0 and YT0, respectively. At the genus level, the top three most abundant genera were Penicillium, Fusarium, and Chrysosporium (Table 5). The relative abundance of Penicillium was increased (*p* < 0.05) in H variety from HT0 to HT2 and then decreased (*p* < 00.05) from HT2 to HT8, whereas increased significantly (*p* < 0.05) from YT0 to YT8 continuously. The relative abundance of Fusarium was not much varied between two groups (H and Y) but it significantly (*p* < 0.05) decreased from YT0 to YT2. The relative abundance of Chrysosporium was not varied significantly (*p* < 0.05) from HT0 to HT8 but it increased (*p* < 0.05) from YT0 to YT2. At the species level, *F. oxysporum, Penicillium abidjanum*, and *Chrysosporium pseudomerdarium* were the top three abundant species (Supplementary Figure 3).

**TABLE 4:** Relative abundance of major phyla of fungi in both varieties *Honghua Dajinyuan* and *Yunyan 87 (Y)* treatments (Mean ± SEM).

Treatment	Ascomycota	Mortierellomycota	unidentified	Basidiomycota	Chytridiomycota
HT0	0.83 ± 0.02^ab^	0.09 ± 0.01^ab^	0.03 ± 0.00	0.03 ± 0.01^b^	0.03 ± 0.01^abc^
HT2	0.79 ± 0.02^ab^	0.05 ± 0.01^bc^	0.07 ± 0.02	0.06 ± 0.01^a^	0.02 ± 0.01^abc^
HT4	0.82 ± 0.02^ab^	0.04 ± 0.01^c^	0.09 ± 0.02	0.01 ± 0.01^b^	0.03 ± 0.02^abc^
HT8	0.88 ± 0.03^a^	0.03 ± 0.00^c^	0.07 ± 0.02	0.01 ± 0.00^b^	0.00 ± 0.00^c^
YT0	0.73 ± 0.03^b^	0.13 ± 0.01^a^	0.05 ± 0.01	0.04 ± 0.02^b^	0.05 ± 0.01^abc^
YT2	0.75 ± 0.03^b^	0.04 ± 0.01^c^	0.07 ± 0.01	0.13 ± 0.03^b^	0.01 ± 0.00^bc^
YT4	0.78 ± 0.02^ab^	0.09 ± 0.00^ab^	0.04 ± 0.00	0.01 ± 0.00^b^	0.07 ± 0.01^a^
YT8	0.79 ± 0.02^ab^	0.06 ± 0.01^bc^	0.01 ± 0.01	0.01 ± 0.00^b^	0.06 ± 0.02^ab^

Values with different superscripts in the same column differ significantly (*P*-value < 0.05).

**TABLE 5:** Relative abundance of major fungal genera in both varieties *Honghua Dajinyuan* and *Yunyan 87 (Y)* treatments (Mean ± SEM).

Treatment	Penicillium	Fusarium	Chrysosporium	Unidentified	Mortierella	Ascomycota	Nectriaceae	Phialophora	Chaetomium	Chytridiaceae	Other
HT0	0.11 ± 0.06^b^	0.12 ± 0.03^a^	0.13 ± 0.07^b^	0.03 ± 0.00	0.09 ± 0.01^b^	0.09 ± 0.02^a^	0.01 ± 0.00^b^	0.01 ± 0.00	0.01 ± 0.00^b^	0.02 ± 0.01^abc^	0.39 ± 0.06
HT2	0.23 ± 0.04^a^	0.10 ± 0.01^ab^	0.11 ± 0.01^b^	0.07 ± 0.02	0.04 ± 0.01^c^	0.04 ± 0.01^ab^	0.03 ± 0.01^b^	0.01 ± 0.00	0.01 ± 0.00^b^	0.02 ± 0.01^abc^	0.30 ± 0.03
HT4	0.06 ± 0.01^b^	0.12 ± 0.02^ab^	0.04 ± 0.01^b^	0.09 ± 0.02	0.03 ± 0.01^c^	0.07 ± 0.01^ab^	0.02 ± 0.00^b^	0.03 ± 0.01	0.02 ± 0.01^b^	0.01 ± 0.00^abc^	0.50 ± 0.08
HT8	0.04 ± 0.02^b^	0.12 ± 0.00^a^	0.10 ± 0.02^b^	0.10 ± 0.02	0.02 ± 0.00^c^	0.05 ± 0.00^ab^	0.10 ± 0.02^a^	0.09 ± 0.05	0.07 ± 0.01^a^	0.00 ± 0.00^c^	0.33 ± 0.02
YT0	0.07 ± 0.01^b^	0.12 ± 0.03^a^	0.08 ± 0.01^b^	0.05 ± 0.01	0.12 ± 0.01^a^	0.06 ± 0.00^ab^	0.01 ± 0.00^b^	0.01 ± 0.00	0.01 ± 0.00^b^	0.04 ± 0.0^abc^	0.42 ± 0.05
YT2	0.10 ± 0.01^b^	0.04 ± 0.01^b^	0.26 ± 0.02^a^	0.07 ± 0.01	0.03 ± 0.01^c^	0.05 ± 0.01^ab^	0.01 ± 0.00^b^	0.03 ± 0.03	0.01 ± 0.00^b^	0.00 ± 0.00^c^	0.40 ± 0.02
YT4	0.12 ± 0.02^ab^	0.14 ± 0.02^a^	0.04 ± 0.01^b^	0.04 ± 0.00	0.08 ± 0.00^b^	0.09 ± 0.02^a^	0.02 ± 0.00^b^	0.01 ± 0.00	0.01 ± 0.00^b^	0.05 ± 0.01^ab^	0.39 ± 0.05
YT8	0.15 ± 0.05^ab^	0.14 ± 0.01^a^	0.04 ± 0.01^b^	0.07 ± 0.01	0.05 ± 0.01^c^	0.03 ± 0.00^b^	0.09 ± 0.03^a^	0.02 ± 0.00	0.06 ± 0.01^a^	0.06 ± 0.02^a^	0.29 ± 0.02

Values with different superscripts in the same column differ significantly (*P*-value < 0.05).

Linear discriminant analysis effect size test was performed to observe the significantly enriched biomarker fungal taxa in both varieties (H and Y) rhizosphere groups (Supplementary Figure 4A). A total of 69 significantly enriched biomarker fungal taxa were detected for both rhizosphere groups (H and Y), where H and Y varieties had 35 and 34 enriched biomarker fungal taxa at different taxonomic levels, respectively. At the genus level, HT0, HT2, HT4, and HT8 were enriched with different biomarker fungi genera (HT0: Chloridium and Ascomycota; HT2: Penicillium; HT4: Entophlyctis and Acrophialophora; and HT8: Sordariomycetes, Sordariales, Chaetomium, Phialophora, and Nectriaceae), whereas YT0, YT2, YT4, and YT8 were found enriched with biomarker fungi genera (YT0: Malbrachea and Mortierella; YT2: Microascus, Chondrogaster, Hysterangiales, and Chrysosporium; YT4: Cordycipitaceae and Trichoderma; and YT8: Capnodiales and Chytridiaceae). Further, the cladogram was constructed to reveal the relative abundance of significantly enriched biomarker fungi taxa from phylum to species level (Supplementary Figure 4B).

### Beta diversity analysis

**FIGURE 1:**
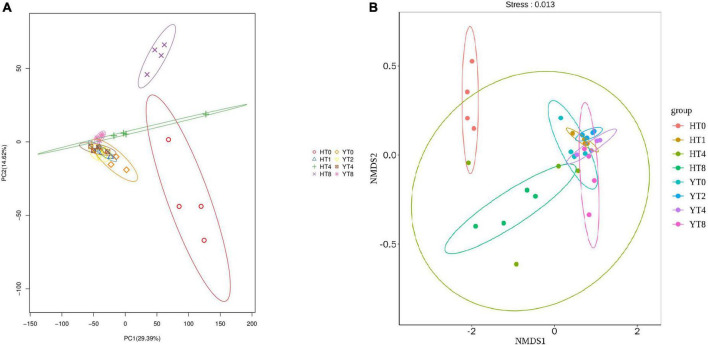
Beta diversity analysis showing distribution of fungal communities among samples of both varieties *Honghua Dajinyuan* (H) and Yunyan 87 (Y) **(A)** Principal component analysis (PCA) analysis and **(B)** Non-Metric Multi-Dimensional Scaling (NMDS) analysis, each treatment group is colored differently and each point in figure show differences between the groups and within group samples.

Principal component analysis was carried out to reveal the similarities and differences among different samples of both varieties (Figure 1A). The PC1 and PC2 explained the 29.39 and 14.62% of total variance, respectively. The Y variety rhizosphere samples (YT0, YT2, YT4, and YT8) were clustered together showing quite similar fungal communities among them, whereas H variety samples (HT0, HT2, HT4, and HT-8) were separated apart showing more differences in fungal communities among them. In comparison, PCA analysis showed that both varieties (H and Y) had different fungal communities. Further, the NMDS analysis also showed the more similar fungal community structure for Y variety treatment groups than H variety treatment groups (Figure 1B).

### Environmental factors affecting the fungal communities

**FIGURE 2:**
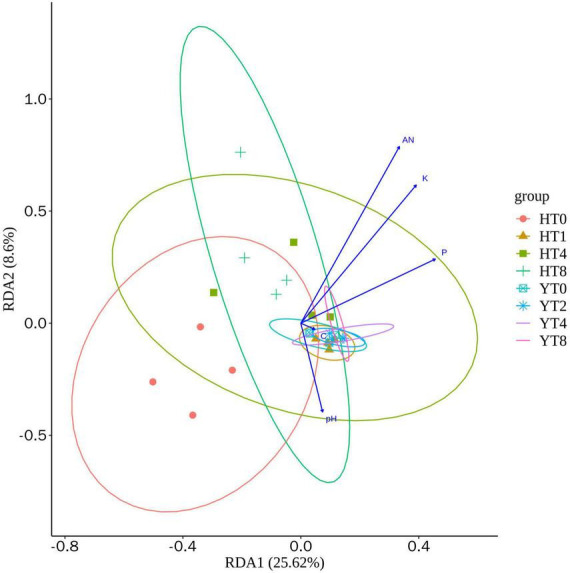
Redundancy analysis (RDA) analysis showing the relationship between environmental factors (AN: alkaline hydrolyzed nitrogen; P: phosphorus; K: potassium; C: carbon; and pH) and fungal communities of both varieties *Honghua Dajinyuan* (H) and *Yunyan 87* (Y).

The redundancy analysis (RDA) was performed to predict the environmental factors (AN; P: phosphorus; K: potassium; C: carbon; and pH) on different rhizosphere soil group samples (Figure 2). The RDA1 explained the 25.62 and 8.6% of total variance, whereas the between the environmental factors and different treatment groups of both varieties (H and Y). The carbon content of rhizosphere soil had more influence on the fungal communities’ structure of Y variety treatment groups, whereas the pH had more influence on H variety treatment groups. Moreover, the AN, P, and K had little effect on the fungal community structure of rhizosphere of both varieties.

### Functional annotation of fungal communities

**FIGURE 3:**
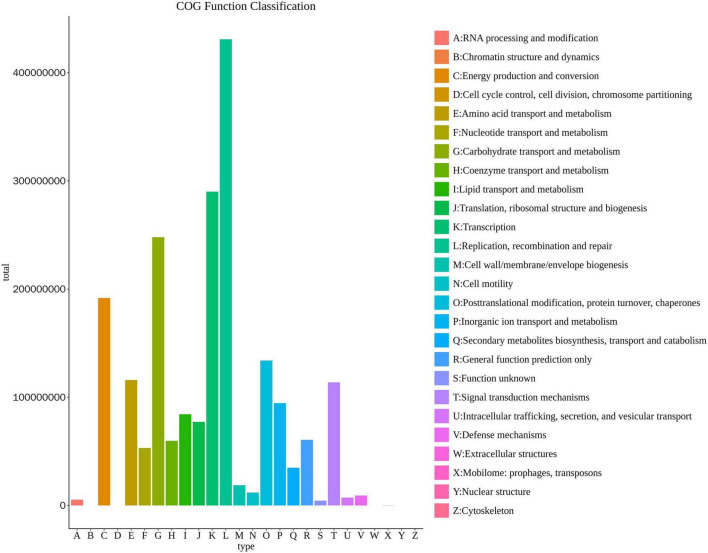
COG function annotation classification histogram. The horizontal axis is the COG function annotation classification, and the vertical axis is the annotation abundance.

The highly enriched (*p* < 0.05) metabolic pathways in *H. Dajinyuan* (H) and *Yunyan 87* (Y) are listed in Table 6. Metacyc database was searched to identify metabolic pathways involved in primary and secondary metabolism of fungal communities of rhizosphere of both varieties (H and Y). The functionally enriched abundant (*p* < 0.05) pathways were Calvin–Benson–Bassham cycle, coenzyme A biosynthesis I, fatty acid β-oxidation I, fatty acid elongation-saturated, glyoxylate cycle, heme biosynthesis I (aerobic), L-leucine degradation I, NAD salvage pathway II, pentose phosphate pathway (non-oxidative branch) (Table 6). The fungal communities in H rhizosphere showed increased (*p* < 0.05) functional abundance from HT0 to HT8, whereas Y variety showed decreased abundance of pathways from YT0 to YT4 and later on increased in YT8. The top three highly enriched pathways in both varieties (H and Y) were fatty acid elongation-saturated, fatty acid β-oxidation I, and glyoxylate cycle pathways. Further, the functional annotation of fungal communities of both varieties was performed using the COG database. The COG database functions and their abundance is presented in form of a histogram (Figure 3). The most abundant functions included replication, recombination and repair, transcription, carbohydrate transport and metabolism, and energy production and conversion.

**TABLE 6:** Relative abundance of functionally enriched metabolic pathways (Mean ± SE) for each treatment group of both varieties *Honghua Dajinyuan* (H) and *Yunyan 87* (Y).

Trt	GlyIII	CBBC	CoAB	FABO	FAES	GlyoxyC	HemeB	LLeuD	NADSP	PPP
HT0	15,290 ± 2,129^b^	21,128 ± 2,464^b^	14,099 ± 1,793^b^	35,860 ± 4,338^b^	50,161 ± 4,745^b^	30,818 ± 3,882^b^	15,027 ± 1,791^b^	11,731 ± 1,548^bc^	9,698 ± 980^b^	24,132 ± 3,154^b^
HT2	18,323 ± 2,008^b^	27,431 ± 2,819^b^	17,232 ± 1,836^b^	41,323 ± 4,256^b^	11,6687 ± 13,616^b^	38,049 ± 4,120^b^	19,566 ± 4,196^b^	21,912 ± 2,126^b^	13,045 ± 1,616^b^	30,905 ± 3,182^b^
HT4	30,532 ± 8,587^b^	41,753 ± 1,1302^b^	28,287 ± 8,008^b^	68,576 ± 18,216^b^	96,266 ± 19,861^b^	64,566 ± 18,930^b^	29,689 ± 8,167^b^	18,861 ± 3,366^bc^	25,576 ± 8,789^b^	46,197 ± 11,384^b^
HT8	61,951 ± 7,733^a^	85,861 ± 9,905^a^	53,752 ± 6,305^a^	14,3311 ± 17,314^a^	24,2349 ± 21,553^a^	12,8752 ± 16,350^a^	59,818 ± 7,115^a^	40,718 ± 3,272^a^	47,409 ± 6,994^a^	94,960 ± 10,815^a^
YT0	24,276 ± 3,622^b^	32,290 ± 5,146^b^	22,309 ± 3,033^b^	56,181 ± 8,722^b^	72,896 ± 13,551^b^	48,148 ± 7,206^b^	24,001 ± 3,707^b^	16,803 ± 3,457^bc^	15,936 ± 2,363^b^	37,489 ± 6,087^b^
YT2	15,257 ± 42,122^b^	20,591 ± 3,046^b^	13,750 ± 1,913^b^	29,839 ± 4,901^b^	49,664 ± 10,733^b^	29,963 ± 4,404^b^	15,521 ± 1,963^b^	14,126 ± 2,005^bc^	10,393 ± 1,795^b^	24,649 ± 3,049^b^
YT4	11,372 ± 3,401^b^	16,398 ± 5,229^b^	10,779 ± 3,370^b^	27,989 ± 8,416^b^	50,345 ± 20,843^b^	23,731 ± 7,496^b^	11,619 ± 3,681^b^	8,900 ± 3,324^c^	8,587 ± 2,598^b^	18,520 ± 5,584^b^
YT8	21,847 ± 7,445^b^	28,805 ± 7,517^b^	19,920 ± 6,974^b^	49,113 ± 1,4753^b^	89,812 ± 13,510^b^	40,678 ± 10,345^b^	22,046 ± 7,086^b^	14,247 ± 1,647^bc^	13,014 ± 2,237^b^	31,139 ± 7,784^b^

Values with different superscripts in the same column differ significantly (*P*-value < 0.05). (Trt, treatment; GlyIII, glycolysis III [from glucose]; CBBC, Calvin–Benson–Bassham cycle; CoAB, coenzyme A biosynthesis I; FABO, fatty acid β-oxidation I; FAES, fatty acid elongation-saturated; GlyoxyC, glyoxylate cycle; HemeB, heme biosynthesis I [aerobic]; LLeuD, L-leucine degradation I; NADSP, NAD salvage pathway II; PPP, pentose phosphate pathway [non-oxidative branch]).

## Discussion

### Physicochemical properties and fungal community structure of soil

Long-term continuous cropping is usually practiced in agriculture, however, it has several adverse effects such as decreased crop production, soil quality, and increased incidence of plant diseases (Du et al., 2022; Guo et al., 2022). The purpose of the present study was to analyze the changes in fungal communities structure, physicochemical properties, and functionally enriched metabolic pathways between two varieties of tobacco, *Yunyan 87* (Y) (high root rot resistant) and *H. Dajinyuan* (H) (susceptible to root rot) under long-term continuous cropping culture. The alpha diversity (ACE, chao1, Simpson index, and Shannon index) and beta diversity matrices revealed significant differences between fungal communities’ structure of both varieties (H and Y) under continuous monoculture with higher fungal diversity in H than Y. Further, the beta diversity analysis revealed H variety rhizospheres had more differences in fungal communities from HT0 to HT8, whereas Y variety rhizospheres exhibited more similar fungal community structure from YT0 to YT8. Lower fungal diversity in resistant variety indicates that the root rot resistant variety had the narrow spectrum of fungal communities that might be attributed to selective breeding that eventually led to more similar fungal communities as reported previously (Kinnunen-Grubb et al., 2020; Favela et al., 2021). Further, the soil physicochemical properties (like AN, AP, AK, and OM) were increased from HT0 to HT8, whereas the Y variety showed an inconsistent effect from YT0 to YT8. However, the soil pH decreased in response to 8 years of monocultures in both varieties. Previously, long-term monoculture of tobacco resulted in the deterioration of soil physicochemical properties (Wang et al., 2017a; Chen et al., 2018). The improvement in physicochemical properties for susceptible variety under long term monocropping might be attributed to its more diverse fungal communities structure than resistant variety, as diverse microbial communities can improve the soil quality (Wang et al., 2020). The other reason could be the use of special fertilizers which can play important role in the sustainability of soil quality (Lal, 2020). The decrease in soil pH (soil acidification) can also result from use of chemical fertilizers (Pahalvi et al., 2021).

The relative abundance of fungal communities revealed Ascomycota, Mortierellomycota, and uncharacterized (unidentified) fungi as top three abundant phyla in both tobacco varieties. For both H and Y, the relative abundance of Ascomycota was increased, whereas the relative abundance of Mortierellomycota was decreased under continuous monoculture. Ascomycota is the largest and most diverse true fungal group found in rhizosphere soils (Arafat et al., 2019; Gao et al., 2019). The majority of Ascomycota are saprophytic, as they decompose OM and induce plant diseases ranging from powdery mildews to rots, malignancies, and vascular wilts (Dong et al., 2022). Previous studies reported that the decrease in Ascomycota abundance was associated with a decreased incidence of Fusarium wilt (Yao and Wu, 2010; Shen et al., 2015). Some Mortierella species have been reported to be hostile for plant diseases such as root rot or potato scab and exhibited disease suppression characteristics (Li et al., 2022). So, the higher abundance of Ascomycota and lower abundance of Mortierellomycota might have played a role in the increased disease incidence rate and disease index observed in susceptible variety under continuous mono-cropping. At the genus level, Penicillium, Fusarium, and Chrysosporium were the top three abundant genera. Relative abundance of Penicillium (*P. abidjanum*) was significantly increased from YT0 to YT8, whereas it increased from HT0 to HT2 and then decreased later up to HT8. Further, Chrysosporium (*C. pseudomerdarium)* relative abundance was increased whereas Fusarium (*F. oxysporum)* was decreased from YT0 to YT2. Some Penicillium species are involved in the production of solubilized phosphorus, siderophore, and phytohormones such as indole acetic acid and gibberellic acid, all of which are beneficial to plant health (Elias et al., 2016; Altaf et al., 2018). Penicillium species have shown to suppress the root rot disease caused by Fusarium species and promote plant growth (Wang et al., 2022a). Fusarium species like *F. oxysporum* and *F. solani* are the major cause of tobacco root rot disease (Yang et al., 2020a; Gai et al., 2021). *Fusarium oxysporum* is one of the most prevalent plant disease, ranking fifth among the top ten plant fungal infections (Dean et al., 2012). Fusarium diseases can enter plant roots and influence vascular tissues, interfering with the transport of critical nutrients from roots to aboveground plant parts. Fusarium infections can generate toxins throughout their growth, development, and metabolism, causing plant wilt and death (Abi Saad et al., 2022). *Chrysosporium pseudomerdarium* has been found to play a positive role in improving plant growth by producing gibberellins (Waqas et al., 2014). In summary, our findings infer that the cultivation of H and Y varieties for continuous years increased the abundance of fungal phyla like Ascomycota while decreasing the Mortierellomycota. Further, fungal taxa mainly Penicillium for longer term and Chrysosporium for first 2 years in Y variety might have played a positive role in managing the pathogenic Fusarium spp. that causes the root rot in tobacco. Furthermore, RDA revealed that carbon content of rhizosphere soil had a higher influence on fungal community structure of Y than H variety, whereas pH exhibited a stronger influence on fungal communities of H variety than Y. Previous studies have also reported changes in fungal community structure by utilization of carbon sources in soils leading to better plant growth (Wang et al., 2016, 2017b). The addition of carbon sources can play role in root rot disease suppression and increased plant growth (Davey et al., 2021).

### Functionally enriched metabolic pathways

Metacyc database was searched to identify functionally enriched metabolic pathways of fungal communities. The functional enrichment of metabolic pathways was increased from HT0 to HT8, mainly attributed to the increased diversity of fungal communities. In contrast, functional abundance in Y rhizospheres decreased from YT0 to YT4 as not much variation was observed in fungal communities’ structure from YT0 to YT4. The top three highly enriched pathways observed in both varieties were fatty acid elongation-saturated, fatty acid β-oxidation I, and glyoxylate cycle. The previous study has shown that rhizosphere microbes release fatty acids to cope with different stresses and to regulate microbial community structure (Bi et al., 2021). These fatty acid metabolic pathways could be involved in the metabolism of different primary and secondary metabolites to cope with stress like disease attacks. Higher relative enrichment of these metabolic pathways in H than Y rhizospheres might be attributed to the increased metabolic response against the higher relative abundance of pathogenic fungal communities or disease stress. Glyoxylate cycle has been found to play important role in tricarboxylic acid (TCA) cycle for gluconeogenesis and more importantly in seedling germination by metabolizing the reserves (Deng et al., 2022). Further, highly abundant COG functions like replication, recombination and repair, transcription, carbohydrate transport and metabolism, and energy production and conversion are also an indication of increased abundance and diversity of fungal communities. So, overall it can be inferred that the higher enrichment of metabolic pathways in H variety might be attributed to the higher metabolic stress due to higher load of pathogenic fungal communities as compared to Y rhizosphere which exhibited lower functional enrichment of these stress pathways (owing to less pathogenic stress) and relatively higher abundance of beneficial fungal communities.

## Conclusion

The present study concluded that both *N. tabacum* varieties *H. Dajinyuan* (root rot susceptible) and *Yunyan 87* (root rot resistant) showed significant differences between their physicochemical properties, fungal communities’ structure, and functional enrichment of metabolic pathways under long-term monoculture cropping. The resistant variety (*Yunyan 87*) had less diverse fungal communities and lower enrichment of functional metabolic pathways that were mainly attributed to long-term selective breeding practices which reshaped microbial diversity. However, the abundance of Penicillium increased under long-term continuous monoculture cropping in resistant tobacco variety than in susceptible variety.

## Data availability statement

The datasets presented in this study can be found in online repositories. The names of the repository/repositories and accession number(s) can be found in the article/Supplementary material.

## Author contributions

JA and YiL: conceptualization. ZW and BL: methodology. YiL, QY, and JA: software. YiL and JA: validation. ZW, QY, BL, and YiL: formal analysis. BL and ZW: investigation. YiL and ZW: resources. QY and YiL: data curation. YiL: writing – original draft preparation. YiL: writing—review and editing. JA: visualization. YiL: supervision. JA: project administration. YiL: funding acquisition. All authors have read and agreed to the published version of the manuscript.

## Footnotes

1
http://cutadapt.readthedocs.io/en/stable/

2
https://github.com/torognes/vsearch/

3
http://www.drive5.com/uparse/

4
http://www.arb-silva.de/

5
http://www.drive5.com/muscle/

6
http://www.r-project.org/
