# Supplementary material for: Differentially enriched fungal communities in root rot resistant and susceptible varieties of tobacco (Nicotiana tabacum L.) under continuous monoculture cropping
Source: Front Microbiol. 2022 Dec 7;13:1036091. doi: 10.3389/fmicb.2022.1036091 (PMC9768445; doi:10.3389/fmicb.2022.1036091)
Supplement: Supplementary file 1 [file Data_Sheet_1.docx]

Supplementary Material


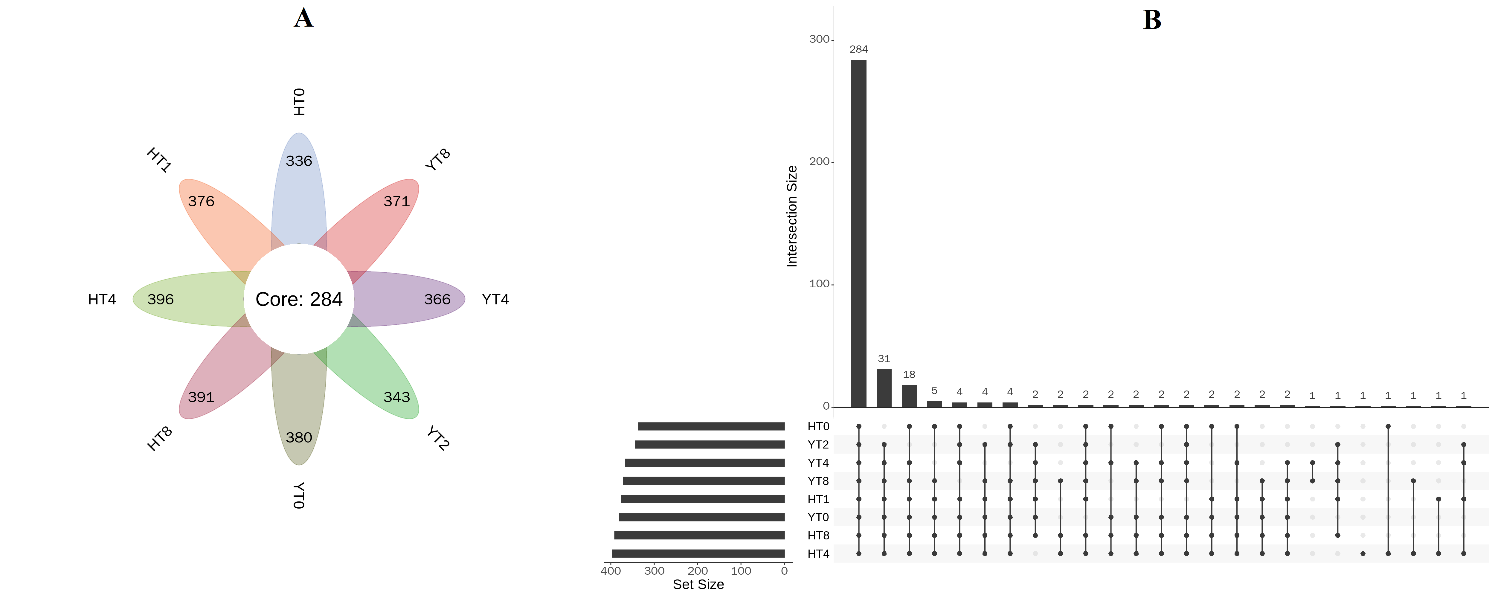


**Supplementary Figure S-1.** **(A)** Intergroup Venn diagram showing unique and common OTUs for each treatment group of both varieties *Honghua Dajinyuan* (H) and *Yunyan 87* (Y). **(B)** Upset plot showing the common OTUs between different treatment groups


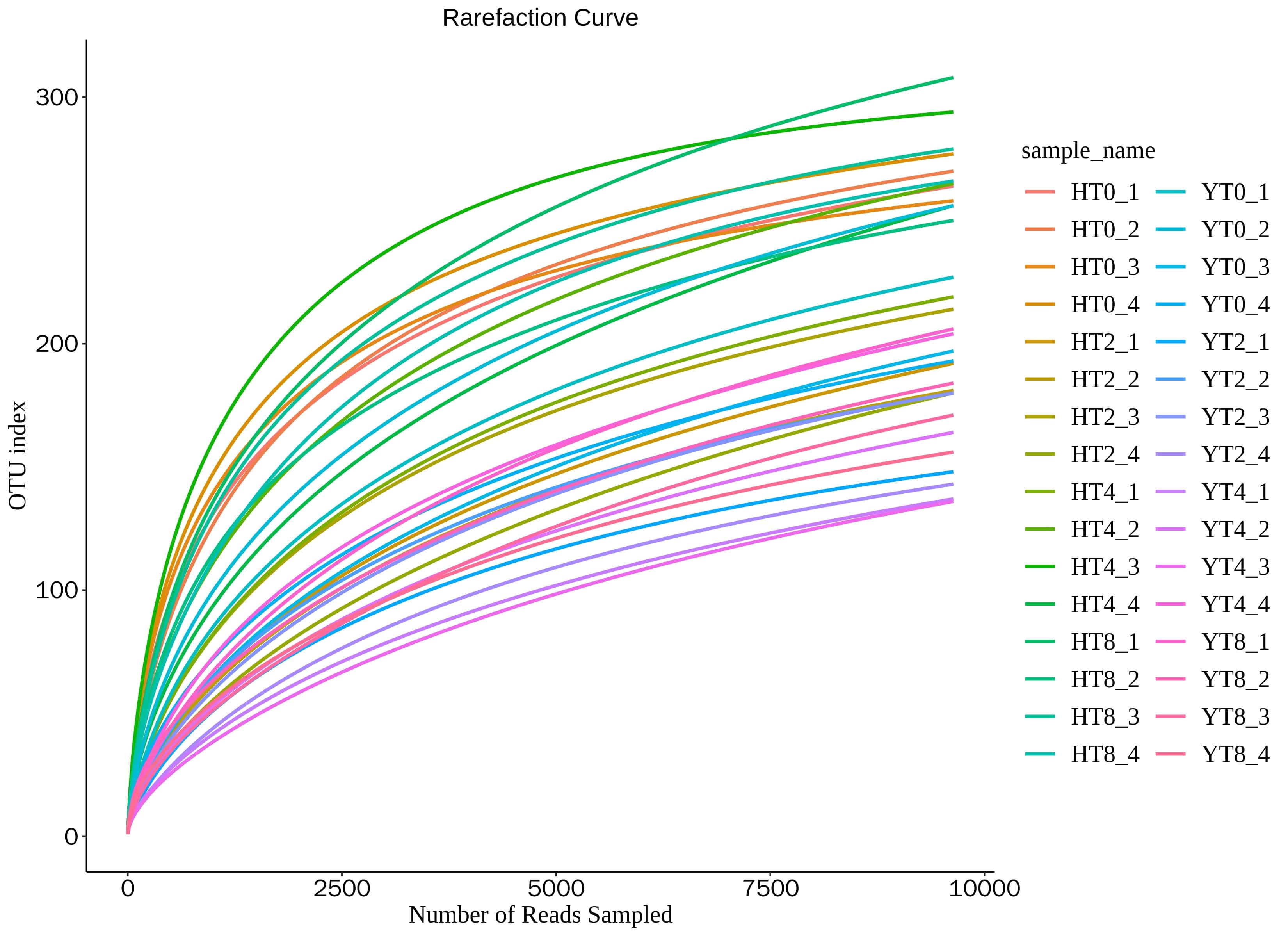


**Supplementary Figure S-2A** Rarefaction curve, horizontal axis showing the number of sequences drawn randomly vs vertical axis showing the observed operational taxonomic units (OTUs) for each treatment group of both varieties *Honghua Dajinyuan* (H) and *Yunyan 87* (Y).


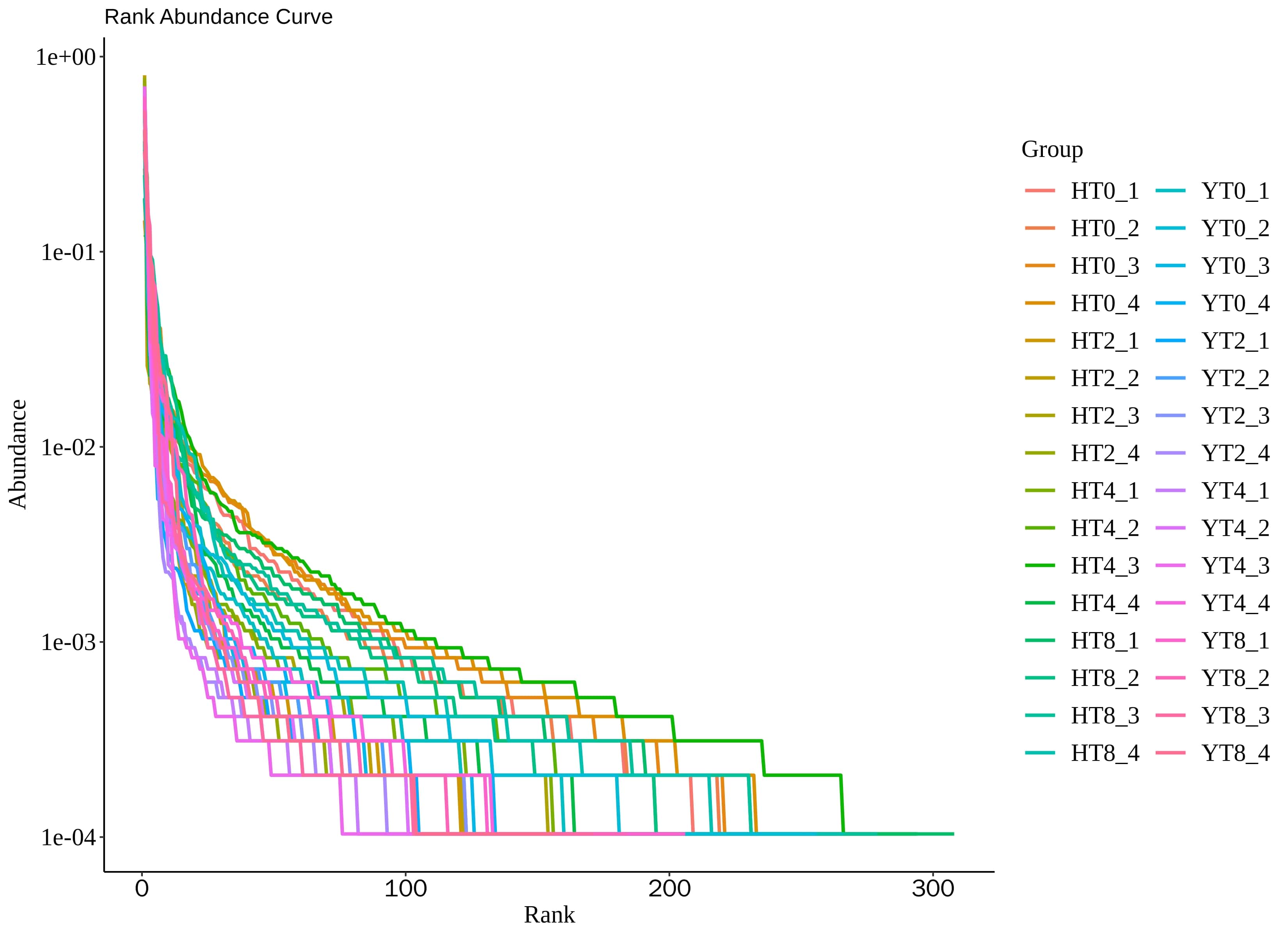


**Supplementary Figure S-2B.** Rank abundance curve showing the relative abundance and species rank for each treatment group of both varieties *Honghua Dajinyuan* (H) and *Yunyan 87* (Y).

**
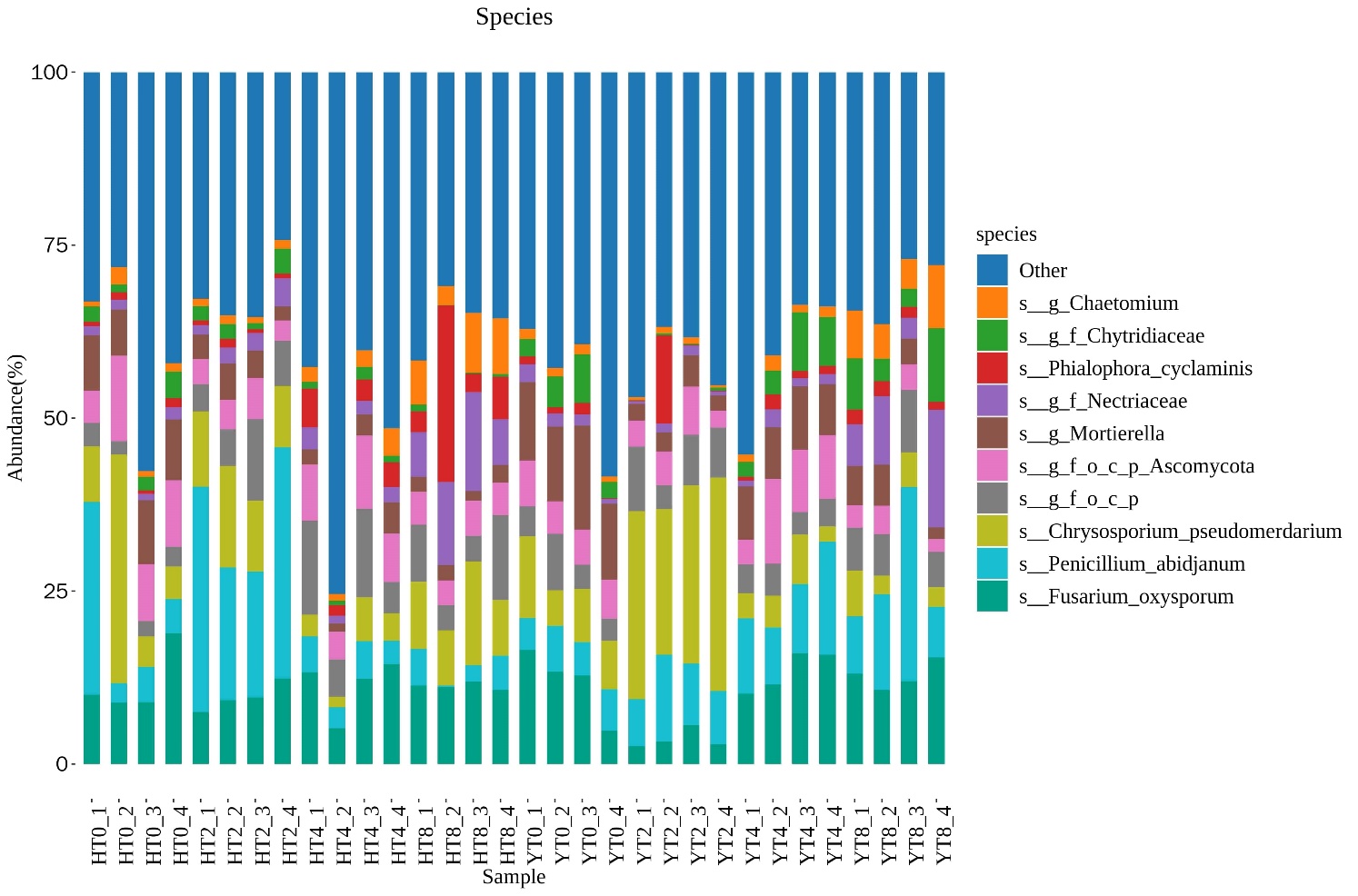
**

**Supplementary Figure S-3.** Relative abundance of fungal communities at species level, for each treatment group of both varieties *Honghua Dajinyuan* (H) and *Yunyan 87* (Y).


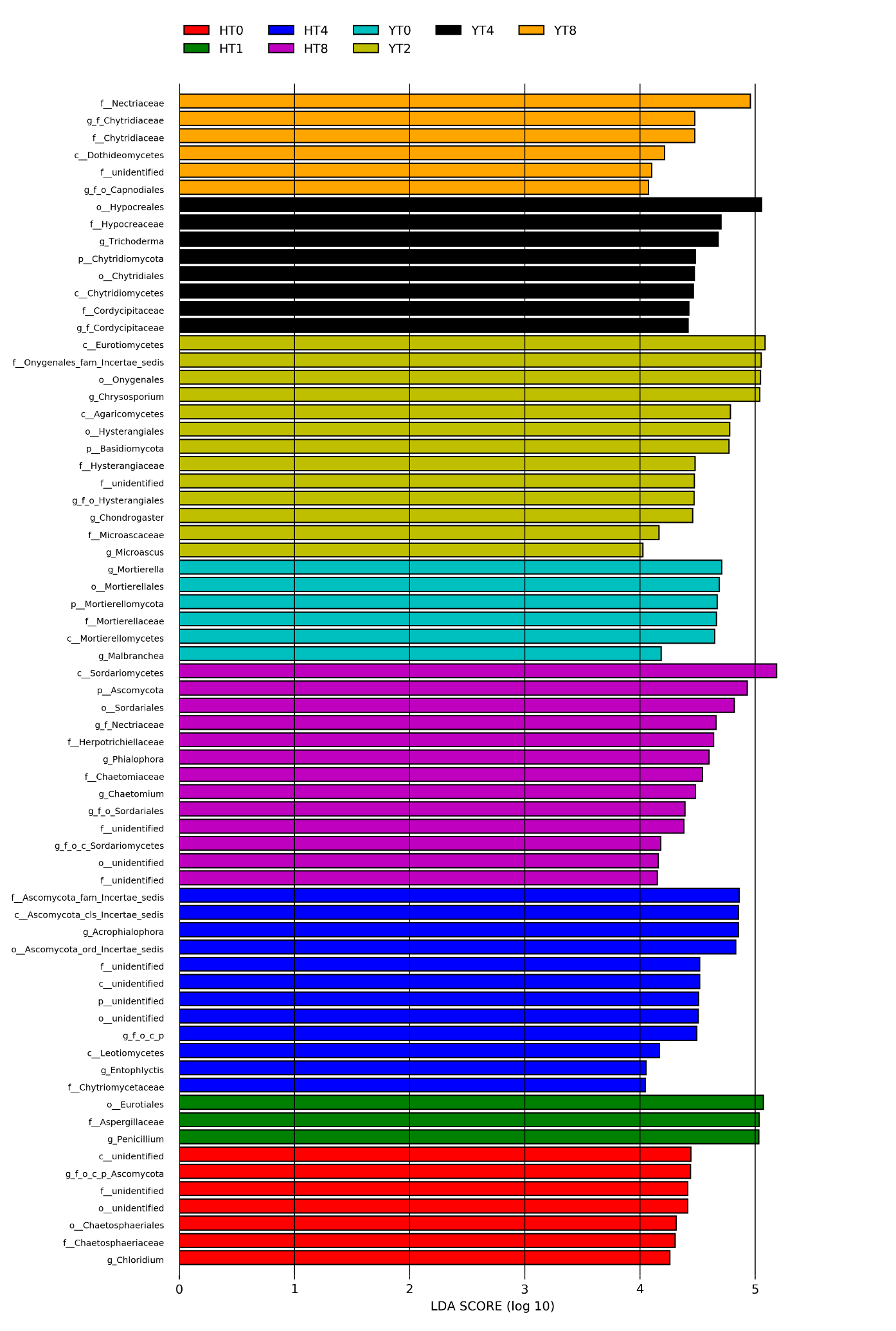


**Supplementary Figure S4-A** Linear discriminant analysis effect size (LEfSe) analysis showing biomarker fungal taxa for samples of both varieties *Honghua Dajinyuan* (H) and *Yunyan 87* (Y) at all taxonomic levels


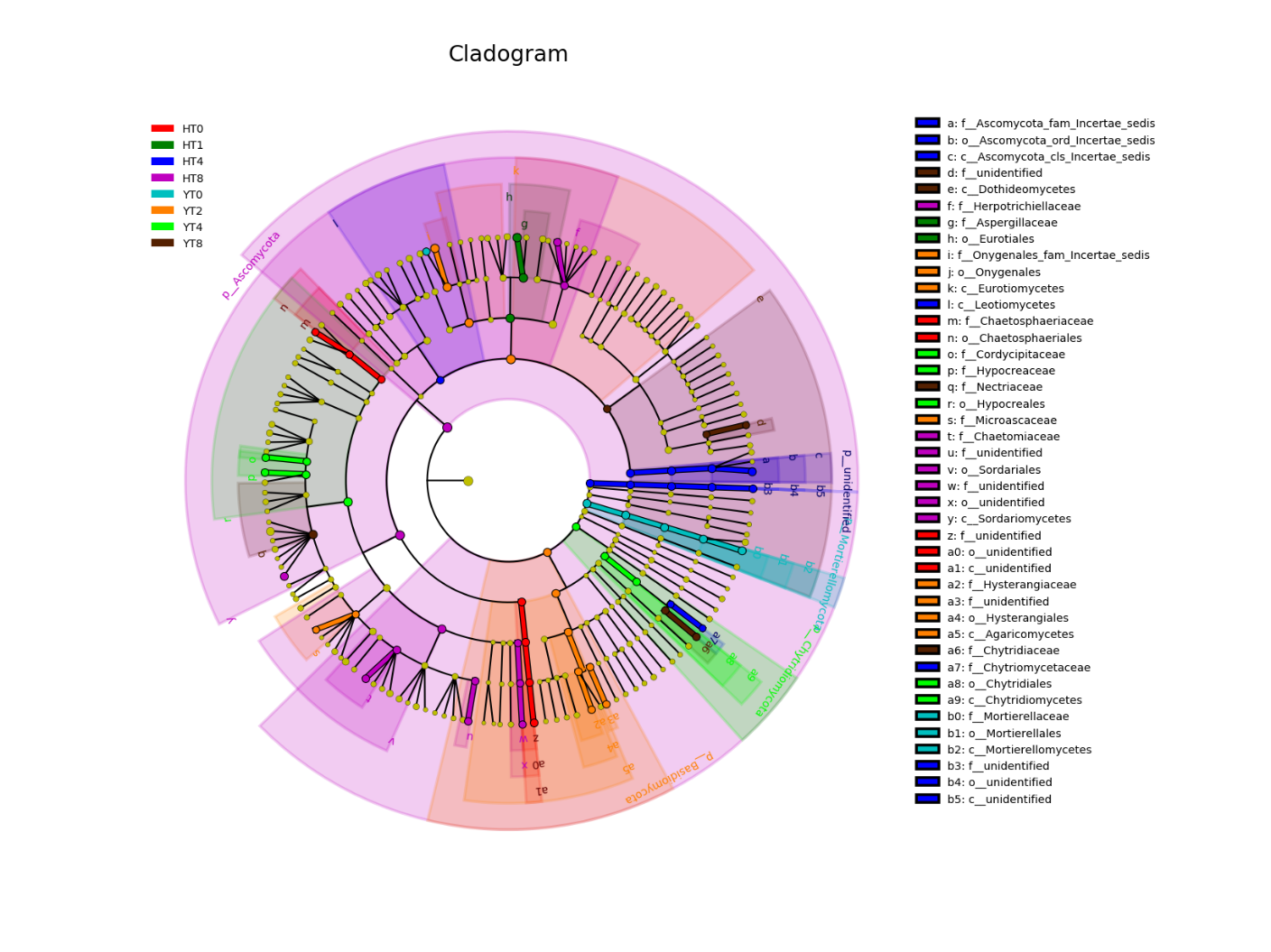


**Supplementary Figure S4-B** Cladogram showing significantly enriched fungal taxa (from phylum to species level) for both varieties *Honghua Dajinyuan* (H) and *Yunyan 87* (Y).

**Supplementary Table S-1.** Information of Both varieties, their treatment and replicates with sample numbers

| **Variety** | **Treatment Name** | **Replicate** | **Sample No** |
| --- | --- | --- | --- |
| *Honghua Dajinyuan* (H) | Control (HT0) | HT0-1 | 1 |
|  |  | HT0-2 | 2 |
|  |  | HT0-3 | 3 |
|  |  | HT0-4 | 4 |
|  | Two years rhizosphere soil (HT2) | HT2-1 | 5 |
|  |  | HT2-2 | 6 |
|  |  | HT2-3 | 7 |
|  |  | HT2-4 | 8 |
|  | Four years rhizosphere soil (HT4) | HT4-1 | 9 |
|  |  | HT4-2 | 10 |
|  |  | HT4-3 | 11 |
|  |  | HT4-4 | 12 |
|  | Eight years rhizosphere soil (HT8) | HT8-1 | 13 |
|  |  | HT8-2 | 14 |
|  |  | HT8-3 | 15 |
|  |  | HT8-4 | 16 |
| *Yunyan 87 (Y)* | Control (YT0) | YT0-1 | 17 |
|  |  | YT0-2 | 18 |
|  |  | YT0-3 | 19 |
|  |  | YT0-4 | 20 |
|  | Two years rhizosphere soil (YT2) | YT2-1 | 21 |
|  |  | YT2-2 | 22 |
|  |  | YT2-3 | 23 |
|  |  | YT2-4 | 24 |
|  | Four years rhizosphere soil (YT4) | YT4-1 | 25 |
|  |  | YT4-2 | 26 |
|  |  | YT4-3 | 27 |
|  |  | YT4-4 | 28 |
|  | Eight years rhizosphere soil (YT8) | YT8-1 | 29 |
|  |  | YT8-2 | 30 |
|  |  | YT8-3 | 31 |
|  |  | YT8-4 | 32 |
